# Prolonged ventricular pause associated with ticagrelor use: A case report

**DOI:** 10.1002/ccr3.5017

**Published:** 2021-10-25

**Authors:** Alaa Rahhal, Amer Aljundi, Sara Saeed Ibrahim Mohamed, Muhammad Awais Arif, Abdul Rahman Arabi

**Affiliations:** ^1^ Pharmacy Department Heart Hospital Hamad Medical Corporation Doha Qatar; ^2^ Internal Medicine Department, Hamad General Hospital Hamad Medical Corporation Doha Qatar; ^3^ Cardiology Department, Heart Hospital Hamad Medical Corporation Doha Qatar

**Keywords:** adverse drug reaction, ticagrelor, ventricular pause

## Abstract

**Case:**

We report a case of a 76‐year‐old female who presented with non‐ST elevation myocardial infarction and developed a 22‐second ventricular pause with ticagrelor that did not recur after shifting to clopidogrel. Based on the Naranjo algorithm, the likelihood that our patient's prolonged ventricular pause was due to ticagrelor exposure was probable.

**Conclusion:**

Ticagrelor use is associated with prolonged ventricular pauses, warranting close monitoring, particularly during the first week of therapy.

## Graphical abstract

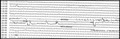

## 1 BACKGROUND

Ticagrelor is an oral reversible antagonist of adenosine diphosphate P_2_Y_12_ receptor which is approved by FDA in the management of acute coronary syndromes (ACS); unstable angina pectoris, ST‐segment elevation myocardial infarction (STEMI) or non‐STEMI, including those managed medically or with percutaneous coronary intervention (PCI), or coronary artery bypass grafting (CABG).^1^

In the PLATO (Ticagrelor versus clopidogrel in patients with acute coronary syndromes) trial,^2^ treatment with ticagrelor compared with clopidogrel significantly reduced the composite endpoint of death from vascular causes, myocardial infarction, or stroke without an increase in the rate of overall major bleeding. Among safety outcomes, ventricular pauses ≥3 s during the first week of therapy were significantly more frequent with ticagrelor than clopidogrel. However, ventricular pauses ≥5 s difference did not reach statistical significance. Following the PLATO trial, there have been emerging case reports of ticagrelor‐associated bradyarrhythmias.^3^, ^4^ Unlike previous cases, we report a case of prolonged ticagrelor‐associated pause (22 s) within 1 h of ticagrelor initiation in a patient without a baseline conduction abnormalities, which required ticagrelor discontinuation and shifting to clopidogrel.

## 2 CASE DESCRIPTION

**FIGURE 1:**
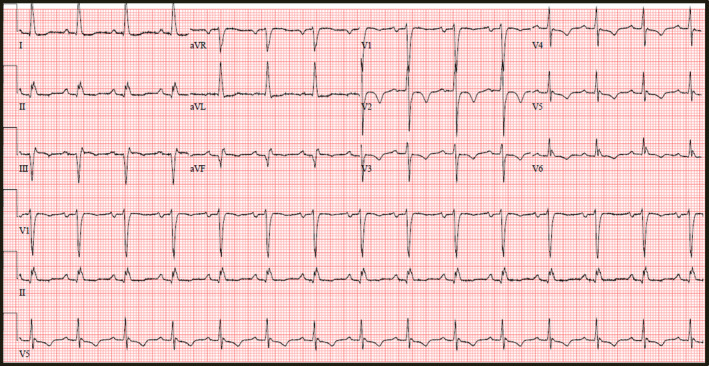
T wave inversion in anterolateral leads (V3–V6)

A 76‐year‐old female without a past history of conduction diseases and confirmed past medical history significant for type 2 diabetes mellitus, hypertension, and coronary artery disease post PCI to right coronary artery (RCA) in 2018, treated with aspirin 100 mg, atorvastatin 20 mg, metoprolol tartrate 50 mg, isosorbide mononitrate 20 mg, and basal‐bolus insulin, presented to our hospital emergency department with a 5‐day history of shortness of breath that was increasing in severity and associated with central chest pain radiating to throat that started 6 h prior to admission. Her vital signs were as follows: blood pressure = 115/73 mmHg, heart rate = 106 beats per minute, temperature = 36.5 C, and oxygen saturation = 95% on room air. Her first 2 sets of troponin T were 104 ng/L and 135 ng/L (normal <14 ng/L), and her transthoracic echocardiogram showed left ventricular regional wall motion abnormalities with an ejection fraction of 38%. Her initial electrocardiogram (ECG) showed T wave inversion in anterolateral leads (V3–V6 leads) as shown in Figure 1. Therefore, a diagnosis of non‐STEMI was made, and she was planned for in‐patient coronary angiography (CAG) and hence started on aspirin 300 mg then 100 mg enteric coated (EC) daily, clopidogrel 300 mg then 75 mg daily, and enoxaparin 1 mg/Kg subcutaneous every 12 h.

On the second day of admission, she underwent CAG and was found to have 95% stenosis in proximal left anterior descending (LAD) artery, and a patent drug‐eluting stent (DES) in the mid‐RCA. A DES (3.0 × 33 mm) was placed in the proximal to mid LAD. After the PCI, she was on aspirin 100 mg EC, clopidogrel 150 mg daily, bisoprolol 5 mg, atorvastatin 40 mg, lisinopril 5 mg, spironolactone 12.5 mg daily, furosemide 40 mg intravenous twice daily, and insulin (basal glargine and bolus aspart). Two days after the PCI, it was decided to start the patient on ticagrelor 180 mg as a loading dose followed by 90 mg twice daily and to stop clopidogrel.

**FIGURE 2:**
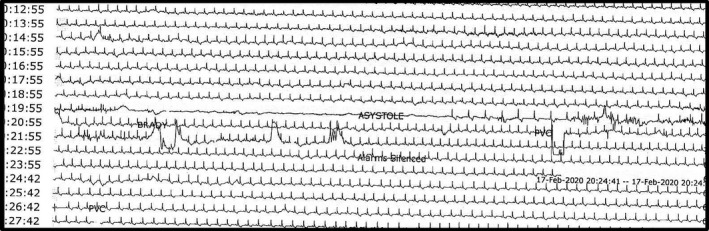
A 22‐s pause on telemetry

One hour after receiving ticagrelor loading dose, the patient started to develop dyspnea followed by an episode of unresponsiveness. The review of the telemetry monitoring strips showed bradycardia followed by a sinus pause of 22 s during the episode of unresponsiveness as shown in Figure 2. Therefore, she was transferred immediately to our cardiac intensive care unit (CICU) where she underwent transvenous pacemaker insertion, which was kept for 2 days, and ticagrelor was switched back to clopidogrel after 24 h of receiving ticagrelor loading dose, by giving a loading dose of 600 mg followed by 75 mg daily. After the removal of the pacemaker, metoprolol 12.5 mg twice daily was started, and she was observed for 2 days and remained stable without any further episodes of bradycardia or sinus pause with a heart rate ranging from 65–75 beats per minute. After which, she was discharged and then seen in the outpatient clinic within 20 days of discharge and had no complaints.

## 3 DISCUSSION

Ventricular pauses are defined as absence of a QRS complex that lasts for more than 2.5 s.^5^ It can be caused by sinoatrial (SA) node dysfunction or atrioventricular (AV) node dysfunction or other causes.^6^

In the phase IIb trial (DISPERSE‐2) that compared 2 regimens of ticagrelor (90 mg twice daily and 180 mg twice daily) to clopidogrel, dose‐related ventricular pauses were more common among patients receiving ticagrelor. Pauses of more than 2.5 s occurred in 4.3%, 5.5%, and 9.9% for clopidogrel, ticagrelor 90 mg [*p* = 0.58], and ticagrelor 180 mg [*p* = 0.0.014], respectively. Pauses of more than 5 s occurred in 0.3%, 1.6% [*p* = 0.22], and 2.1% [*p* = 0.06], respectively.^5^

Because of this observation, the investigator of PLATO trial excluded patients at increased risk for symptomatic bradyarrhythmias (such as patients with known sick sinus syndrome, second‐degree or third‐degree AV block, or previous syncope likely to be due to bradycardia unless a pacemaker was in place) and included a prospectively designed continuous ECG monitoring to determine the incidence of ventricular pauses with ticagrelor and identify any associated symptoms. During the first week of treatment, ventricular pauses of 3 s or more occurred in 5.8% of patients on ticagrelor compared with 3.6% of patients on clopidogrel (RR: 1.61; 95% CI: 1.14–2.26), and pauses of 5 s or more occurred in 2.0% of patients on ticagrelor compared with 1.2% of patients on clopidogrel (RR: 1.66; 95% CI: 0.92–3.01). The difference was mainly due to an excess in sinoatrial (SA) node pauses in ticagrelor group. Ventricular pauses were mostly asymptomatic and nocturnal.^6^

Compared with clopidogrel, ticagrelor has been shown to increase adenosine plasma concentration in acute coronary syndrome patients by inhibiting adenosine uptake by erythrocytes.^7^ Adenosine is known to suppress the automaticity of cardiac pacemaker cells.^8^ This could explain the potential of ticagrelor to cause adenosine‐mediated effects including ventricular pauses.^6^, ^9^

In the present case, ticagrelor caused 22‐second ventricular pause within the first day of initiation. Although ventricular pauses can be attributed to coronary artery disease itself, we believe that ticagrelor was the main cause of these pauses for several reasons; first, the longer pause of 22 s occurred 2 days after successful revascularization; second, the pause did not recur after shifting to clopidogrel. Moreover, the use of the Naranjo algorithm also indicated a probable relationship between the administration of ticagrelor and the development of prolonged ventricular pause (score of 7).^10^

A challenging decision in our case was whether a loading dose of clopidogrel is required or not in a patient who had received a loading dose of ticagrelor. Following the SWAP‐4 (switching From ticagrelor to clopidogrel in patients with coronary artery disease) trial that compared 3 different strategies of clopidogrel dosing (600 mg loading dose 12 h after last ticagrelor dose followed by 75 mg daily, 600 mg loading dose 24 h after last ticagrelor dose followed by 75 mg daily, and 75 mg daily without a loading dose), 600 mg clopidogrel was given 12 h after the last dose of ticagrelor followed by clopidogrel 75 mg daily.^11^

In comparison to previously reported cases of ticagrelor‐induced pauses, our patient who was already tolerating beta‐blocker prior to admission, developed a longer pause after a very short time of ticagrelor initiation. Nicol et al. reported an 8‐s ventricular pause in a 39‐year‐old male with STEMI within 1 h of receiving ticagrelor loading dose and atenolol 25 mg.^3^ And Low et al. reported that a 59‐year‐old female who presented with non‐STEMI developed four pauses, the longest was 18.5 s in duration, within 3 h of initiating ticagrelor.^4^

## 4 CONCLUSION

Ticagrelor use is associated with prolonged ventricular pauses. Thus, patients who are receiving ticagrelor should be monitored closely, particularly during the first week of therapy. If ticagrelor‐induced ventricular pauses occur and a decision is made to shift to clopidogrel, a loading dose of 600 mg should be administered to maintain an acceptable level of platelet inhibition and prevent major cardiovascular events.

## ACKNOWLEDGEMENTS

Open access funding provided by Qatar National Library.

## CONFLICT OF INTEREST

The authors declare that there is no conflict of interest.

## AUTHOR CONTRIBUTIONS

AR contributed to study design conception, literature review, acquisition of relevant data, writing the first draft of the manuscript, and approving the final version of the manuscript; AA contribute to the literature review, acquisition of relevant data, and approving the final version of the manuscript; SSIM contributed to writing the case description and approving the final version of the manuscript; MAA and ARA contributed to study design conception and approving the final draft of the manuscript.

## CONSENT

A written informed consent for publishing patient's information and images was obtained from the patient.

## Data Availability

The datasets used and/or analyzed during the current study are available from the corresponding author on reasonable request.
